# Developing Asthma in Childhood from Exposure to Secondhand Tobacco Smoke: Insights from a Meta-Regression

**DOI:** 10.1289/ehp.10155

**Published:** 2007-06-28

**Authors:** Kathleen L. Vork, Rachel L. Broadwin, Robert J. Blaisdell

**Affiliations:** Air Toxicology and Epidemiology Branch, Office of Environmental Health Hazard Assessment, California Environmental Protection Agency, Oakland, California, USA

**Keywords:** childhood asthma, environmental tobacco smoke, ETS, meta-analysis, meta-regression, relative risk, secondhand tobacco smoke, SHS

## Abstract

**Objective:**

Studies have identified associations between household secondhand tobacco smoke (SHS) exposure and induction of childhood asthma. However, the true nature and strength of this association remains confounded in many studies, producing inconsistent evidence. To look for sources of potential bias and try to uncover consistent patterns of relative risk estimates (RRs), we conducted a meta-analysis of studies published between 1970 and 2005.

**Data sources:**

Through an extensive literature search, we identified 38 epidemiologic studies of SHS exposure and the development of childhood asthma (that also controlled for atopy history) from 300 potentially relevant articles.

**Data synthesis:**

We observed substantial heterogeneity within initial summary RRs of 1.48 [95% confidence interval (CI), 1.32–1.65], 1.25 (1.21–1.30), and 1.21 (1.08–1.36), for ever, current, and incident asthma, respectively. Lack of control for type of atopy history (familial or child) and child’s own smoking status within studies and age category altered summary RRs in separate meta-regressions. After adjusting for these confounding characteristics, consistent patterns of association emerged between SHS exposure and childhood asthma induction. Our summary RR of 1.33 (95% CI, 1.14–1.56) from studies of incident asthma among older children (6–18 years of age) is 1.27 times the estimate from studies of younger children and higher than estimates reported in earlier meta-analyses.

**Conclusions:**

This new finding indicates that exposure duration may be a more important factor in the induction of asthma than previously understood, and suggests that SHS could be a more fundamental and widespread cause of childhood asthma than some previous meta-analyses have indicated.

Asthma in childhood becomes a lifelong condition for many people, and there is evidence that its prevalence has increased over the past 50 years (Ross Anderson et al. 2007). Direct and indirect severe impacts on general health, well-being, and premature death can lead to large costs to the health care system for the management and treatment of asthma. The role of secondhand tobacco smoke (SHS) in asthma exacerbation is well accepted [U.S. Department of Health and Human Services (DHHS) 2006], whereas its role in childhood asthma induction is less well understood. Several reviews and meta-analyses have weighed the evidence of a causal relationship between exposure to SHS and the onset of asthma in children [Cook and Strachan 1999; Office of Environmental Health Hazard Assessment (OEHHA) 1997; Strachan and Cook 1998; U.S. DHHS 2006; U.S. Environmental Protection Agency (EPA) 1992].

These reviews and meta-analyses differ in their conclusions about the sufficiency of evidence to infer a causal relationship between SHS exposure and asthma induction in children. The U.S. EPA and California EPA concluded that SHS exposure is causally associated with an increase in the incidence of childhood asthma (OEHHA 1997; U.S. EPA 1992), based on studies of young children. Strachan and Cook (1998) observed elevated estimates of relative risk (RRs) from studies of preschool age and equivocal RRs from studies of older children. Their meta-analysis, originally conducted in the mid-1990s, was updated in the most recent Surgeon General’s Report (SGR) on *Health Effects from Involuntary Exposure to Tobacco Smoke* (U.S. DHHS 2006). The SGR concluded that the evidence is suggestive, but not sufficient to infer a causal relationship between SHS and the induction of childhood asthma. The SGR’s main reason for this conclusion is that the small number of studies that examined an association of SHS exposure from parental smoking with asthma incidence among older children (when there is reasonable diagnostic certainty) found inconsistent evidence of elevated RRs.

Many additional individual epidemiologic studies have been published since 2001 (the cutoff of Strachan and Cook’s latest update in the SGR) (Gilliland et al. 2001; Hessel et al. 2001; Jaakkola et al. 2001; Kivity et al. 2001; Mannino et al. 2001; Pokharel et al. 2001; Sturm et al. 2004; Takamura et al. 2001). Several of these newer studies have reported that control for important confounding variables such as atopy history (family or child history of atopy), prenatal exposure to maternal smoking, and smoking status of older children can substantially alter RRs (Gilliland et al. 2001; Hessel et al. 2001; Jaakkola et al. 2001; Pokharel et al. 2001). Because atopy history was a particularly important confounder, we chose to conduct a meta-analysis of studies that controlled for atopy history. We examined the effects of other potentially confounding factors and study-wide characteristics on the summary RR of developing childhood asthma from exposure to household SHS to see whether consistent patterns of RRs emerge.

One of our goals was to use meta-regression on atopy-controlled studies to quantitatively explore the effects of these other potentially important sources of heterogeneity on the summary RR. Some of the heterogeneity among RRs reported in previous meta-analyses (Cook and Strachan 1999; Strachan and Cook 1998; U.S. DHHS 2006) may be related to uncontrolled confounding factors such as atopy history, age, sex, race, and the status of smoking in older study subjects. Other sources of heterogeneity among studies may include the age at which exposure and disease status are assessed, the assessment of maternal smoking versus other SHS sources, the evaluation of prenatal versus postnatal SHS exposure, the use of asthma incidence versus asthma prevalence as the outcome measure, the type of study design, or the subject recruitment source.

Our systematic review considered studies addressed in the meta-analyses of Cook and Strachan (1999), OEHHA (1997), Strachan and Cook (1998), and U.S. DHHS (2006). Our meta-analysis used the power provided by the more recent studies and the simultaneous examination of multiple characteristics through meta-regression to further examine the relationship between SHS exposure and induction of childhood asthma. We were particularly interested in the relationship between household SHS exposure and childhood asthma induction in older children.

## Methods

### Literature search

We requested a literature search for SHS as a risk factor in the development of childhood asthma examined in epidemiologic studies published between 1970 and 2005. Outcome key words included asthma, wheezy bronchitis, asthmatic bronchitis, and reactive airway disease. Exposure key words included ETS, environmental tobacco smoke, passive smoking, secondhand smoke, involuntary smoke, tobacco smoke pollution, and cigarette smoke. We limited the search to asthma in childhood and adolescence (through 18 years of age).

A professional experienced librarian searched PubMed (http://www.pubmed.gov), Web of Science (http://portal.isiknowledge.com/), Biosis Previews(http://portal.isiknowlege.com/), Toxline (http://toxnet.nlm.nih.gov), Scifinder Scholar (http://www.cas.org/products/sfacad/index.html), Environmental Sciences and Pollution Management (http://www.csa.com), Melvyl (http://melvyl.cdlib.org), WorldCat (OCLC) Firstsearch (http://firstsearch.oclc.org), University of California and San Francisco Tobacco Control Archives (http://www.library.ucsf.edu/tobacco/). Manual searches were conducted from review articles and previous meta-analyses. When necessary, we contacted authors for additional information or for translations from languages other than English.

### Selection criteria

We developed eight criteria to select studies from among peer-reviewed articles. First, studies should present *a*) outcomes of the development (not exacerbation) of new cases of asthma or, because of ambiguities related to the diagnosis of asthma in young children, wheezy bronchitis (but not wheeze alone); we used broad criteria to define new incident cases of asthma that included both allergic and nonallergic descriptions of asthma. However, we sought to include all studies that identified cases of asthma in their analyses by other criteria than symptoms of wheeze alone. Our definition included asthma, wheezy bronchitis, or asthma/wheeze that was ever or currently recognized by doctor diagnosis or by a set of symptoms that are recognized criteria for diagnosing asthma in addition to wheezing.

We also included asthma identified through parental response to pilot-tested or standardized questionnaires on respiratory health. Studies should also present *b*) comparable groups of subjects (i.e., exposed and unexposed, cases and referents selected by the same criteria); *c*) at least one source of postnatal household SHS exposure; *d*) adequate data for extracting or calculating RRs and their standard errors; this information may be presented as odds or rate ratios or estimates of relative risk; *e*) results for children (0–18 years of age) where the child was the unit of analysis; *f* ) completed and original work (not abstracts of work in progress or reviews); *g*) reports written in languages other than English that are commonly spoken in Europe (i.e., German, Italian, French, and Spanish); and *h*) studies that controlled for the confounding effect of atopy history because atopy has such a strong association with asthma and parental smoking behavior. Our control for atopy history category included: family history of allergy or asthma; childhood diagnosis of allergic conditions other than asthma, such as eczema, hay fever, or allergic rhinitis; the respondent in the study reported symptoms of allergic conditions; or the study investigator stratified on an indicator of atopy such as skin-prick test results. We considered the study controlled for atopy history if the study restricted the selection of subjects to children with, subgroups were stratified by, or the estimate of RR was statistically adjusted for atopy history as defined above. We rejected studies that did not meet these criteria. When more than one analysis was conducted on the same set of children, we included information from multiple articles if they provided unique information about the children.

### Data abstraction

We extracted RRs and standard error estimates from publications using methods described by Greenland (1987). Odds ratios were corrected to RRs even though the prevalence of asthma was often not much more than 10% in the study population (Zhang and Yu 1998). We initially analyzed studies from which population prevalence estimates were not available (i.e., from many case–control studies), and later removed them from the pool of available studies.

The variables we considered as potential covariates in each meta-regression are described in Table 1. We grouped covariates into two subgroups and gave each covariate a value of 0 or 1. We considered that a study controlled for a covariable if the study restricted the selection of subjects to children from a single covariable category, or if the subgroups were stratified by, or the estimate of RR was statistically adjusted to, persons who belong to a single category of that covariable. We classified SHS exposure as average (or adjusted to 15 cigarettes/day smoked in the home).

### Data analysis

The first two meta-analyses in this review are an analysis of average SHS exposure on RRs from studies of newly diagnosed or persistent asthma (current asthma), and the RR from studies of asthma that may have occurred early in life and subsequently resolved (ever asthma). In the third meta-analysis of SHS effect on asthma incidence in this review, we included studies that examined SHS exposure effects and classified subjects by continued exposure before the onset of asthma.

Where possible, we collapsed exposure levels within studies that used a common reference group and adjusted for correlations among estimates using methods described by Greenland and Longnecker (1992). We adjusted exposure measures such as cotinine levels in blood and the number of smokers in the home or cigarettes smoked by household occupants to be approximately equivalent to the number of cigarettes smoked per day in the home [see Supplemental Material, Appendix A for the details and conversion factors used for this adjustment (http://www.ehponline.org/docs/2007/10155/suppl.pdf)].

Although most of the RRs in the studies included in this review indicated a positive association for both current and ever asthma, there was substantial heterogeneity in the magnitude of the SHS effect across studies. We performed separate subanalyses for studies of current and ever asthma because we suspected that differences in study design and case detection specific to the type of asthma studied might explain RR heterogeneity. Within cohort studies that classified subjects by exposure status at the start of follow-up, we sought to examine the effect of exposure years on incident asthma. Each analysis is comprised of independent sets of study subjects. Several studies provided separate estimates of RR for both current and ever asthma. To avoid overlapping populations among analyses, we included only the current asthma RR.

We estimated summary RRs using inverse variance-weighted least-squares methods (DerSimonian and Laird 1986). If the chi-squared statistic from the homogeneity test was greater than its degrees of freedom, we used a random-effects method to account for between-study variability; otherwise, we used a fixed-effects method.

To explore the sources of RR heterogeneity, we modeled the log RR as a function of predictors in a linear meta-regression model. Each covariate was considered as a potential modifier of the RR. We included all covariates for which the *p*-value was < 0.1.

We regarded the model as accounting for RR heterogeneity if the homogeneity chi-square was less than or equal to its degrees of freedom. If our model did not account for the heterogeneity in this sense, after considering all eligible covariates, we examined the influence of individual studies. We detected outliers by removing one study at a time and estimating a weighted average estimate of all other studies. We conducted this study influence analysis using “Metainf,” a statistical function of Stata version 8.0 (StataCorp. LP, College Station, TX) that sequentially removes one study at time, and then recalculated RR to determine the effect that the removed study’s RR has on the average RR for all studies.

We managed data using SAS version 8.2 (SAS Institute Inc., Cary, NC) and Microsoft Office Excel 2000 ( Microsoft, Redmond, WA) and analyzed the data using Stata (version 8.0).

## Results

**Figure 1:**
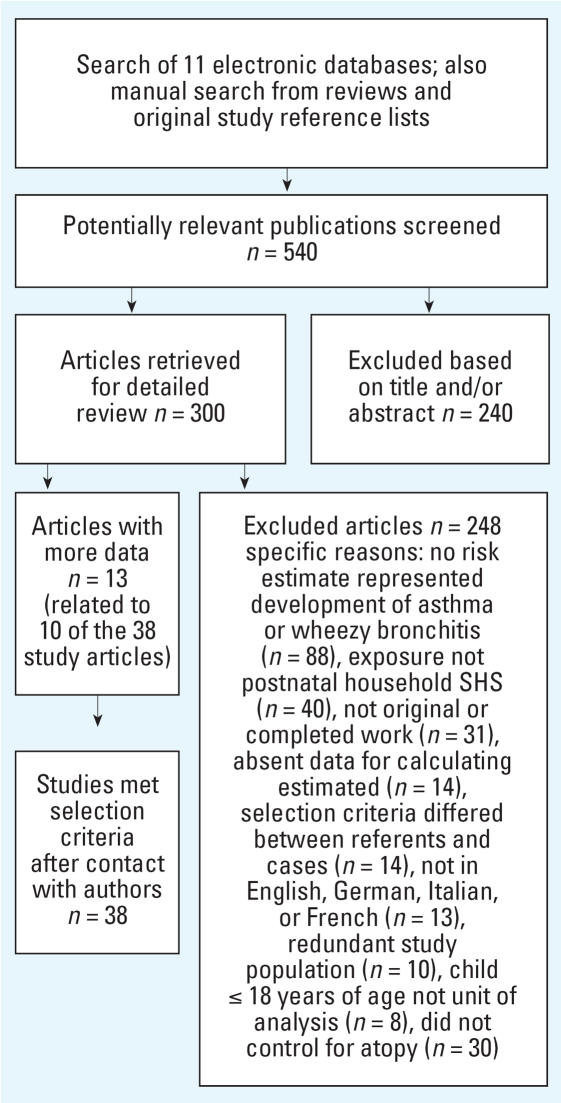
Literature search strategy.

Our search generated > 500 abstracts for review, which produced 300 potentially relevant articles. We received responses from six of the eight authors we had contacted to obtain additional information needed to determine a study’s eligibility. Thirty-eight articles reported at least one risk estimate that met our inclusion criteria (Tables 2–4). Twelve articles [Aberg et al. 1995; Aberkane 1998; Al-Frayh et al. 1992; Bener et al. 1993a, 1993b; Bergmann et al. 1998; Franklin et al. 1985; Luttmann et al. 1993; Moussa et al. 1996; Medical Research Council (MRC) 1966, 1986; Ronmark et al. 1999] and the Abacci Atlas website (Shank 2000; http://www.abacci.com/atlas/demography.asp?countryID=352) provided information on additional characteristics about the study design or population associated with 10 of these studies. We rejected 248 articles; in cases where there were multiple reasons for exclusion, we have reported only one (see Figure 1 for details).

We treated each of the 38 articles as an individual study unless RRs were reported for more than one independent set of study subjects. For example, because Chen et al. (1996) reported distinct estimates for children by atopic history, this study was subdivided into two studies. This process produced 53 studies total. Of these, 32 were cross-sectional, 13 were case–control, and 8 were cohort designs.

### Study methodologies and key characteristics

Tables 2–4 describe the 53 studies meeting the eight criteria that we have included in our meta-analyses. These studies include approximately 200,000 children and adolescents (≤18 years of age) from 20 countries.

### Study design

About half of the studies that examined average household SHS exposure and current asthma were case–control and half were cross-sectional design. Studies ranged from < 200 to > 100,000 study subjects. All but one of the studies that examined average household SHS exposure and ever asthma were cross-sectional. Among the cohort studies, populations ranged from< 200 to nearly 15,000 subjects.

### Summary RR using different exposure reference groups

The summary or weighted average of RRs, tests for RR heterogeneity, and RR percentiles are shown in Table 5 for each meta-regression. Before combining estimates of RR, we applied a correction factor to odds ratios and removed five studies (four current asthma, one ever asthma) that did not provide an estimate of prevalence. Among studies of current asthma, a homogeneous (*p* = 0.528) combined estimate of RR emerged. The observations we included in our corrected analyses are included in online tables (Supplemental Material, available online at http://www.ehponline.org/docs/2007/10155/suppl.pdf). Substantial heterogeneity among cross-sectional studies was observed in the ever asthma group when the reference group was no household exposure, and among the incident asthma group when the reference group was no maternal smoking exposure. Results are presented from random-effects models. RRs tended to be similar in eight cohort studies for which the SHS exposure was assessed before the onset of asthma [summary RR = 1.21; 95% confidence interval (CI), 1.08–1.36; RR range, 0.84–1.49] to 11 case–control studies or cross-sectional studies of average household SHS exposure and current asthma where exposure to SHS was assessed at the time of diagnosis or case identification (summary RR = 1.25; 95% CI, 1.21–1.30; RR range, 0.86–2.17). The summary RRs among prevalence studies was higher among 23 studies of ever asthma, at 1.48 (95% CI, 1.32–1.65; RR range, 0.57–5.81) (Table 5).

### Household SHS exposure and ever asthma

The observations we included in our subgroup analyses of average household SHS exposure and ever asthma are summarized in Table S1 (Supplemental Material, available online at http://www.ehponline.org/docs/2007/10155/suppl.pdf). Substantial heterogeneity remained (homogeneity *p* < 0.001) after fitting a meta-regression model that included four covariates and for which no additional covariates entered the model. Most of this heterogeneity appeared to be attributed to the summary estimate from one large study (Takemura et al. 2001). We treated this study as an outlier because it used a stricter definition of asthma to classify subjects as cases compared with the other studies included in our database. Classifying subjects in this way might have resulted in a bias downward if milder cases of asthma among children exposed to SHS were classified as noncases. After removing this outlier study, three covariates then accounted for heterogeneity in the meta-regression model: studies that were not *a*) controlled for family history of atopy, *b*) restricted to nonsmoking children, *c*) restricted to children of school age (Table 6).

The strongest modifier of the ever asthma summary RR was lack of control for a child’s own smoking habits. The study summary RRs from studies that did not control for this covariate were 1.35 times higher than controlled studies. Lack of control for *a*) family history of atopy, *b*) age category of the study subjects, and *c*) smoking habits among study subjects were weaker modifiers for ever asthma studies [summary RRs were 0.84 and 1.20 times the joint reference estimate of RR of 1.21 (95% CI, 1.17–1.26); see Table 6 for reference categories]. This estimate is lower than the corresponding summary RR without adjustment for covariates, 1.48 (95% CI, 1.32–1.65; Table 5).

### Household SHS exposure and incident asthma

The observations we included in our subgroup analyses of household SHS exposure and incident asthma are summarized in online Table S2 (Supplemental Material, available online at http://www.ehponline.org/docs/2007/10155/suppl.pdf).

The results of the meta-regression for eight cohort studies appear at the end of Table 6. SHS exposure and incident asthma RR was 0.79 times higher among studies of preschool children than among studies of school-age children, and 0.82 times higher when studies did not control for child atopy than for studies that controlled for this covariable.

The predicted RR for SHS exposure effect on incident asthma among children in the joint reference category of all covariates was 1.33 (95% CI, 1.14–1.56; see Table 6 for reference categories). This estimate is higher than the corresponding summary RR without adjustment for covariates, 1.21, (95% CI, 1.08–1.36; Table 5).

## Discussion

This meta-analysis extends previous work by focusing on atopy-controlled studies, which has been well established as a source of confounding in individual studies, and examining the relationship between SHS exposure and onset of childhood asthma. We have evaluated and adjusted for additional sources of heterogeneity among summary RR by meta-regression methods and observed consistent patterns of elevated RRs among studies. We observed a consistent, positive association between household SHS exposure and current, ever, and incident asthma. We also observed an elevated summary RR for incident asthma that was statistically significant among studies of schoolchildren with postnatal exposure to SHS. We did not find that restricting exposure to postnatal SHS was an important modifier of summary RRs for any of the asthma outcomes. Recent reports by Cook and Strachan (1999) and the U.S. DHHS (2006) have concluded that prenatal exposure to maternal smoking is not necessary to elicit an adverse effect. Our meta-regressions support this conclusion.

In our meta-regression of average household SHS exposure and ever asthma, covariate control accounted for much of the heterogeneity. The effect of not controlling for family history of atopy reduced the RR among ever asthma studies, and lack of control for both child and family history of atopy reduced the estimate of RR among incident asthma studies. These findings suggest that this confounding variable biases the estimate of RR toward the null.

Recent studies have identified elevated levels of endotoxin in cigarette smoke and SHS indoor spaces (Hasday et al. 1999; Larsson et al. 2004). It is plausible that elevated endotoxin exposure would cause elevated immunoglobulin E levels in families (including the child) exposed to SHS, and thus affect the likelihood of atopy and subsequent asthma. However, we were unable to analyze the relationship between exposed and unexposed family members and their atopy (and subsequent asthma) status separately because we did not have individual-level data.

We observed that summary RRs from studies that examined ever asthma among younger children were slightly higher than RRs from studies of exclusively older children. These findings are contrary to the findings from cohort studies but consistent with previous reviews (Cook and Strachan 1999; Strachan and Cook 1998).

Most of the cross-sectional studies assessed SHS exposure status by asking if household or parental sources currently smoked. One explanation of these findings is that RRs from cross-sectional studies of older children could be biased downward because assessment of current SHS exposure status may not reflect early-life exposure. For example, parental smoking habits may change once symptoms of allergy or asthma appear in their children. If this occurred among older asthmatics, then these children may be classified as nonexposed, which would result in a differential misclassification of exposure among cases. This source of bias is avoided in prospective cohort study designs.

When studies did not restrict subjects to nonsmoking children, summary RRs were higher, possibly due to bias upward from exposure among studies that overlooked this source of SHS exposure. It is also possible that study subjects who themselves are smokers are more likely to have been exposed to higher levels of household SHS (Wang et al. 1999). Therefore, including smokers in the analysis would increase the RR of developing asthma among the study population. However, most studies did not assess whether asthma developed among study subjects before taking up a smoking habit of their own.

The RR for the average household SHS exposure effect on ever asthma appeared similar to the effect on current and incident asthma, according to our meta-regression analysis. The RR for prevalent asthma could be slightly lower because of bias downward if household exposure to SHS occurred up to the time of asthma diagnosis and then stopped. This would mean that asthmatics with past but not current household SHS might be misclassified as nonexposed.

Cook and Strachan (1999), Strachan and Cook (1998), and the U.S. DHHS (2006) observed a stronger RR for incident asthma or wheezing illness among younger children compared with their findings among older children. These investigators suggested that the stronger relationship with younger children might be attributed to exacerbation of intercurrent infection among young children, resulting in transient wheeze that would tend to diminish with age and increasing airway caliber. This proposed mechanism would suggest that SHS may not be a sole primary cause of early childhood asthma; rather, it may be one of several required co-factors that when combined may lead to asthma development.

In our cohort study meta-regression, we also found that much of the observed heterogeneity was accounted for by the age category of children in the study. Most important, older children exposed to SHS were more likely than younger children to develop asthma. We found no evidence that RRs from studies using physician diagnosis to identify cases were systematically different than RRs from studies that did not identify cases in this way. The differences in our meta-analysis findings versus earlier studies are likely attributed to differences in approach and the inclusion of studies in our meta-analysis published after the previous meta-analyses. We looked more precisely at the relationship between SHS exposure and asthma as distinct from wheeze alone, and we restricted our analysis to studies that controlled for atopy history. Thus our meta-analysis examines a subtly different question than earlier meta-analyses (Cook and Strachan 1999; Strachan and Cook 1998), and takes advantage of the increased body of epidemiologic literature.

Because cohort studies classify subjects by exposure at the start of follow-up, it is less likely that exposure misclassification would occur and more likely that the child’s age is a reasonable surrogate measure of the length of time study subjects are exposed to household SHS. This positive relationship between a surrogate of duration of household SHS exposure and RR was also observed within individual studies (Neuspiel et al. 1989; Wolf-Ostermann et al. 1995). Hence, a finding that higher summary RRs occurred in studies of older children suggests that SHS exposure duration may also play a fundamental role in the development of asthma.

The positive relationship with age category identified in these studies suggests that the risk of developing asthma from a longer time of exposure to SHS increases in later childhood. The RRs among studies that examined incident asthma were more positively associated with SHS exposure than were studies of prevalent asthma. The elevated RRs for the association between household SHS and prevalent as well as incident asthma suggest that the association between SHS exposure and asthma is not caused by selection or misclassification biases.

A mechanistic theory consistent with our findings holds that the development of asthma can be causally associated with the chronic effects from exposure to SHS on bronchial hyperreactivity rather than the acute effects of SHS exposure on airway caliber (Halken et al. 1995; U.S. EPA 1992). Others have also recently drawn this conclusion [“postnatal exposure shows a causal link with the development of asthma in childhood” (Gilmour et al. 2006); and “strong evidence also supports a causal role of environmental tobacco smoke in childhood asthma, especially in the induction of asthma, but also in the poor overall control of an established disease” Jaakkola and Jaakkola 2002)]. The question of asthma incidence increasing with age and duration of exposure is important because if RR increases with age and duration of exposure, it becomes less likely that asthma from SHS exposure early in life is attributed solely to a transient vulnerability in early childhood that is less likely to result in enduring disease. It also means that public health interventions to stop childhood SHS exposure may have a positive benefit in preventing asthma induction beyond early childhood.

## Conclusions

Clearer understanding of the role of SHS in childhood asthma induction can help inform public health interventions to prevent childhood SHS exposure and the benefits from such interventions. Our analysis attempted to address some of the remaining concerns posed by previous reviewers who stated that the evidence for an association between household SHS exposure and new-onset asthma is equivocal especially among older children. We observed a positive and consistent pattern of association between household SHS exposure and the RR of developing asthma during childhood in our meta-analyses. In contrast to earlier findings, this association was not limited to younger children, certain high-risk populations, or prevalent cases. Similar to previous analyses, we did not find that prenatal exposure to SHS was necessary to observe elevated summary RRs for any of the asthma outcomes.

## Figures and Tables

**Table 1:** Covariates considered in meta-regression based on outcome definition.

	Meta-regression by asthma outcome		
Covariate	Current	Ever	Incident
Geographic region	Yes	Yes	Yes
Age category	Yes	Yes	Yes
Type of SHS source	Yes	Yes	No
Timing of exposure (postnatal only)	Yes	Yes	Yes
Source of study subjects	Yes	Yes	Yes
Type of study	Yes	Yes	No
Not controlled for age or sex	Yes	No	Yes
Not controlled for race	Yes	Yes	No
Not controlled for child’s own smoking	Yes	Yes	No
Not controlled for child history of atopy	Yes	Yes	Yes
Not controlled for family history of atopy	Yes	Yes	No
Not controlled for family and child atopy	Yes	Yes	No
Asthma not identified by doctor diagnosis	Yes	Yes	Yes

“Yes” indicates that the variable was evaluated in meta-regression; “No” indicates that the variable was not evaluated in meta-regression because no observations differed for this covariate or the variable was redundant to another variable.

**Table 2:** Descriptions of the 15 studies and 14 published articles that presented data on household SHS exposure and current asthma for the meta-analyses.

Reference (subgroup)	Study location	Study design	Case doctor diagnosed	No. of cases/ referents	RR	95% CI
Agabiti et al. 1999 (te)	Italy	CC	No	1,060/12,304	1.18	1.03–1.34
Agabiti et al. 1999 (gr)	Italy	CC	No	733/11,811	1.32	1.14–1.54
Azizi et al. 1995	Malaysia	CC	Yes	158/201	1.91	1.13–3.21
Chen et al. 1996 (ac)	Canada	XS	Yes	64/280	0.86	0.39–1.89
Daigler et al. 1991	USA	CC	Yes	137/246	1.96	1.10–3.47
Ehrlich et al. 1996	S. Africa	CC	No (a/w)	325/250	1.32	1.03–1.70
Gilliland et al. 2001	USA	XS	Yes	511/5,048	1.48	1.01–2.17
Mannino et al. 2001	USA	XS	Yes	3,629^a^	1.36	0.88–2.08
Murray and Morrison 1990 (ac)	Canada	CC	Yes	163/233	0.93	0.60–1.60
Palmieri et al. 1990 (nac)	Italy	CC	Yes	72/433	1.47	0.98–2.21
Ronmark et al. 1998	Sweden	XS	No	182/3,249	1.60	1.25–1.95
Selcuk et al. 1997	Turkey	XS	Yes	303/5,109	1.28	0.94–1.75
Soto-Quiros et al. 1994	Costa Rica	XS	No	563/1,865	1.53	1.15–2.03
Sturm et al. 2004	USA	XS	Yes	11,378/92,730	1.24	1.19–1.29
Wolf-Ostermann et al. 1995	Germany	XS	Yes	4,678^a^	1.43	0.96–2.12

Abbreviations: ac, atopic children; a/w, asthma/wheeze; CC, case–control; gr, 6- to 7-year-old subjects; nac, nonatopic children; RR, relative risk estimate (not corrected); te, 13- to 14-year-old subjects; XS, cross-sectional; Yes/No, doctor-diagnosed asthma.

a Study total (case/reference information was not given in the article).

**Table 3:** Descriptions of the 30 studies and 22 published articles that presented data on household SHS exposure and ever asthma for the meta-analyses.

Reference (subgroup)	Study location	Case doctor diagnosed	Cases/ referents	RR	95% CI
Azizi and Henry 1991	Malaysia	Yes (a/w)	206/1,295	1.1	0.9–1.4
Bener et al. 1991	Saudi Arabia	Yes	235/2,806	1.77	0.80–3.91
Burchfiel et al. 1986 (bo)	USA	No	187/1,028	2.16	1.39–2.93
Chen et al. 1996 (ac)	Canada	Yes	76/268^a^	1.39	0.60–3.21
Chen et al. 1996 (nac)	Canada	Yes	17/531	5.82	1.60–21.1
Gergen et al. 1998	USA	Yes	442/7,236	1.45	1.17–1.79
Gilliland et al. 2001	USA	Yes	4,314^a^	1.1	0.9–1.4
Goren and Hellman 1991	Israel	No	870/7,389	1.24	1.05–1.43
Gortmaker et al. 1982	USA	No	188/2,884	1.49	1.08–2.06
Hajnal et al. 1999	Switzerland	No	407/4,034	1.20	0.94–1.54
Jenkins et al. 1993	Australia	No (a/w)	1,349/7,182	1.26	1.12–1.40
Kay et al. 1995	England	No	73/122	1.92	1.27–2.90
Kivity et al. 2001 (Ar,bo,ac)	Israel	No	38/242^b^	1.73	1.44–2.07
Kivity et al. 2001 (Ar,bo,nac)	Israel	No	38/242^b^	1.74	1.12–2.69
Kivity et al. 2001 (Ar,g,ac)	Israel	No	27/351^b^	1.73	1.43–2.09
Kivity et al. 2001 (Ar,g,nac)	Israel	No	27/351^b^	1.61	0.96–2.68
Kivity et al. 2001 (J,bo,ac)	Israel	No	43/244^b^	1.73	1.45–2.06
Kivity et al. 2001(J,bo,nac)	Israel	No	43/244^b^	1.74	1.22–2.50
Kivity et al. 2001 (J,g,ac)	Israel	No	36/262^b^	1.73	1.44–2.07
Kivity et al. 2001 (J,g,nac)	Israel	No	36/262^b^	1.75	1.14–2.69
Le Roux et al. 1995	France	Yes	99/1,094	1.79	1.06–3.02
Maier et al. 1997	USA	Yes	106/819	1.6	0.9–2.7
Mannino et al. 2001	USA	Yes	3,629^a,c^	1.19	0.83–1.72
Pokharel et al. 2001	India	No (a/w)	40/80^d^	3.33	1.85–7.65
Rasanen et al. 2000	Finland	Yes	179/4,399	1.48	0.97–2.25
Ronmark et al. 1998	Sweden	No	276/3,155^a^	1.29	0.95–1.74
Selcuk et al. 1997	Turkey	Yes	888/4,524^a^	1.35	1.12–1.62
Soyseth et al. 1995	Norway	No	51/567	2.8	1.3–6.1
Takemura et al. 2001	Japan	Yes	2,315/21,513	0.95	0.87–1.03
Wolf-Ostermann et al. 1995	Germany	Yes	4,678^a,c^	1.43	0.96–2.12

Abbreviations: ac, atopic children; ap, parents with atopy history; Ar, Arab; a/w, asthma/wheeze; bo, boys; g, girls; J, Jewish; nac, nonatopic children; nap, parents with no atopy history; RR, relative risk estimate (not corrected); Yes/No, doctor-diagnosed asthma.

a Excluded study already included in the current asthma analysis (see Table 5).

b Includes atopic and nonatopic group.

c Study total (case/reference information was not given in the article).

d Case–control study design (all other studies are cross-sectional design).

**Table 4:** Descriptions of the eight studies and eight published papers that presented data on household SHS exposure and incident asthma for the meta-analyses.

Reference (subgroup)	Study location	Case doctor diagnosed	Cases/ referents	RR	95% CI
Bergmann et al. 2000	Germany	Yes	92/788	1.32	0.88–1.97
Jaakkola et al. 2001 (nap)	Norway	Yes	80/1,571	0.84	0.53–1.34
Neuspiel et al. 1989	Britain	No (wb)	590/8,760	1.49	1.18–1.87
Ponsonby et al. 2000	Australia	No	88/205	1.09	0.94–1.26
Sigurs et al. 1995	Sweden	Yes	12/128	1.2	0.41–3.60
Strachan et al. 1996	Britain	No	2,665/11,906	1.10	0.76–1.60
Withers et al. 1998	Britain	Yes	498/1,694	1.50	1.14–1.98
Zeiger and Heller 1995 (ap)	USA	Yes	50/115	1.37	0.55–3.45

Abbreviations: ap, parents with atopy history; nap, parents with no atopy history; RR, relative risk estimate (not corrected); wb, wheezy bronchitis; Yes/No, doctor-diagnosed asthma.

**Table 5:** Summary estimates, 95% CIs and data descriptions for household SHS exposure comparisons grouped by type of asthma.^a^

			Asthma type		Percentile			
Type of combined estimate of RR^b^	No.	RR	95% CI	*p*-Value	Min	25th	75th	Max
Current								
Uncorrected	15	1.30	1.22–1.39	0.249*	0.86	1.24	1.60	2.17
Corrected^c^	11	1.25	1.21–1.30	0.528	0.88	1.23	1.38	2.17
Ever								
All studies^c^,^d^	23	1.48	1.32–1.65	<0.001*	0.57	1.45	1.74	5.81
Household SHS	17	1.51	1.31–1.75	<0.001*	0.94	1.47	1.73	5.81
Maternal smoking	6	1.29	1.15–1.45	0.190*	1.21	1.21	1.98	2.77
Incident								
All studies	8	1.21	1.08–1.36	0.225*	0.84	1.08	1.33	1.49
Household SHS	4	1.13	0.89–1.44	0.544	0.84	0.92	1.26	1.27
Maternal smoking	4	1.24	1.06–1.45	0.074*	1.08	1.08	1.45	1.49

Abbreviations: CI, confidence interval; Max, maximum value of the distribution; Min, minimum value of the distribution; *p*-value, value from the *p* distribution for the null hypothesis that the rate ratio is constant across studies.

a We report random effects estimates.

b We report corrected estimates unless otherwise indicated.

c We removed four studies from the analysis of current asthma (Azizi et al. 1995; Daigler et al. 1991; Murray and Morrison 1990; Palmieri et al. 1990) and one study from the analysis of ever asthma (Pokharel et al. 2001) for lack of enough information to convert odds ratios into estimates of RR.

d Excluded six studies already included in the current asthma analysis.

* Heterogeneity.

**Table 6:** Summary estimates for household SHS exposure comparisons grouped by type of asthma cases [ever (1 outlier excluded) versus incident asthma].^a^

Covariable influence on reference estimate of RR	Combined RRs^b^	95% CI
Ever asthma model^c^		
Joint reference^d^ (*n* = 22)	1.21	1.17–1.26
Not controlled for family atopy (*n* = 12)	1.02	0.90–1.16
Not adjusted for child’s own smoking (*n* = 12)	1.63	1.54–1.73
Includes subjects < 6 years of age (*n* = 5)	1.45	1.39–1.52
Incident asthma model^e^		
Joint reference^f^ (*n* = 8)	1.33	1.14–1.56
Not controlled for child atopy (*n* = 3)	1.09	0.93–1.28
All study subjects < 6 years of age (*n* = 2)	1.05	0.94–1.16

RR, corrected estimate of relative risk.

a We report fixed-effects estimates; the log rate ratio regressed on the set of covariates listed in the table for each meta-regression.

b Ratio of corrected relative risk estimates are comparing studies/strata in the designated index category with studies/strata in the reference category of that covariate.

c Residual homogeneity, *p* = 0.85.

d Ever asthma model reference category = studies that included only older children, controlled for child’s own smoking, and controlled for family atopy.

e Residual homogeneity, *p* = 0.82.

f Incident asthma model reference category = only older children and controlled for child atopy.
